# Early-Life Nutrition in Preterm Infants and Risk of Respiratory Infections and Wheezing: A Scoping Review

**DOI:** 10.3390/nu15133031

**Published:** 2023-07-04

**Authors:** Chiara Nava, Anna Di Gallo, Andrea Biuso, Irene Daniele, Gianluca Lista, Pasquale Comberiati, Diego Peroni, Gian Vincenzo Zuccotti, Enza D’Auria

**Affiliations:** 1 Department of Pediatrics, Buzzi Children’s Hospital, 20154 Milan, Italyandrea.biuso@unimi.it (A.B.);; 2 Division of Neonatology, Buzzi Children’s Hospital, 20154 Milan, Italy; 3 Department of Clinical and Experimental Medicine, Section of Paediatrics, University of Pisa, 56126 Pisa, Italy; 4 Department of Biomedical and Clinical Sciences L. Sacco, University of Milan, 20154 Milan, Italy

**Keywords:** early-life nutrition, wheezing, respiratory tract infection, preterm infants

## Abstract

Introduction: Preterm birth is a common early-life event that can lead to long-term consequences. The incidence of wheezing, asthma, and respiratory tract infections is higher in children born prematurely than in the general population. The purpose of this review was to synthesize the existing literature on the role of early-life nutrition in the later risk of respiratory morbidities. Methods: A scoping review of the literature was performed by searching three online databases. Inclusion criteria were: infants born <37 GWk, comparing human milk versus any other type of milk feeding formulation. Our primary outcomes were wheezing or asthma or respiratory tract infections after discharge. Two authors independently screened the results and extracted study characteristics using a predefined charting form. Results: Nine articles were included (eight cohort studies and one randomized trial). Four studies supported the protective effect of breastfeeding on wheezing or respiratory infections or both. Four studies did not confirm this association. One study confirmed the protective role of breastfeeding only on the subgroup of girls. There was a high heterogeneity among the included studies, in the type of milk feeding, outcomes, and age at follow-up. Conclusions: The current evidence is conflicting. The high heterogeneity and methodological flaws could have influenced the results of the studies. Carefully designed studies are required to define the role of early-life nutrition among preterm infants on their long-term respiratory outcomes.

## 1. Introduction

Preterm birth is a common early-life event, the adverse consequences of which may affect the entire life course. Worldwide, one in ten babies is born preterm, a percentage that continues to rise in most countries. Given the increasing burden of prematurity and the increasing rates of survival among preterm infants, a focus on understanding and limiting its long-term consequences is mandatory.

Respiratory problems are very frequent in this population due to the combination of structural and functional lung immaturity, especially when birth happens at the lowest gestational ages [1]. In these cases, long-term ventilatory support, infections, hyperoxia, pulmonary edema, and inflammation can affect the premature lungs and generate lung injury and persistent pulmonary abnormalities [2]. Nevertheless, moderately preterm infants, who are considered relatively healthy when compared with early preterm neonates and usually need little to no respiratory support in the neonatal period, could develop respiratory problems later on as well [3,4].

While the short-term respiratory complications of prematurity are well known, less is known about the consequences of prematurity on pulmonary function later in life. Many studies and an important meta-analysis [5,6], including 1.5 million children from across six continents, demonstrated that the risk of developing wheezing during childhood is 1.71 times higher for premature infants than for term-born infants (1.46 when adjusted for confounding factors). The risk seems to be proportional to the degree of prematurity, with children born very prematurely bearing a risk three times higher compared to full-term infants [5,6]. A systematic review with a meta-analysis showed that the forced expiratory volume in 1 s (FEV1) was decreased in preterm-born survivors compared with term-born controls at 5 to 23 years old, even in those who did not develop bronchopulmonary dysplasia (BPD) [7]. While previous studies have focused on this association solely during childhood [8,9], some recent studies seem to confirm the association between prematurity and respiratory diseases in adulthood, in particular asthma and chronic obstructive pulmonary disease (COPD) [10,11].

Other than airway obstruction, hyperresponsiveness, and wheezing or asthma, preterm infants have a higher incidence of respiratory tract infections than term infants. Indeed, while respiratory tract infections play a major role in morbidity and hospitalization among infants in general [12], morbidity in preterm infants is even higher: this is due to functional and structural lung immaturity, with “simplified” alveolar structures and a small caliber of airways [13], but also because of the immaturity of the immune system. Serum IgG levels at birth are significantly lower in preterm babies compared to term infants and directly proportional to gestational age (GA), as a consequence of the interruption of the maternal–fetal transfer across the placenta during the last trimester [14,15]. These levels fall during the first three months of life, and only after this period do they start to rise back up, reflecting the infant’s own production, while still not reaching the levels registered in full-term infants [14]. While immunoglobulin paucity determines a higher risk of infections, at the same time the immaturity of the cellular immunity—the lower quantitative and qualitative function of neutrophils—can contribute to increased susceptibility to infections by intracellular pathogens after birth [16].

The immaturity of humoral and cellular immunity, together with altered lung development, leads to a higher risk of infection, in particular to a higher susceptibility to lower respiratory tract infections (LTRIs) and re-hospitalization for infections, especially in early childhood [14,16]. As a consequence of this, the use of antibiotics for respiratory tract infections is higher during the first 4 years of life of a preterm baby, both in early and in moderate prematurity, compared with term newborns [4].

It is worth mentioning the role of respiratory syncytial virus (RSV) in acute lower respiratory infections, which has a large burden of disease worldwide in children, causing 2% of deaths in children aged 0–60 months and 3.6% in children aged 28 days to 60 months [17]. Preterm infants are known to be at increased risk for severe RSV infection, with a hospitalization rate that is estimated to be almost twice that of term infants, even for those born moderately preterm [18,19]. Moreover, it has been proved that the development of RSV-LRI in infancy in preterm infants is associated with an increased prevalence of early childhood wheezing between ages 2 and 3 years [20].

Given the importance of respiratory sequelae of prematurity during the first years of life, the question arises as to what measures can be taken in order to improve this outcome.

Early-life nutrition is one of the most important and studied modifiable factors that can influence later outcomes [21]. Recognizing the role that different aspects of nutrition can have on the respiratory function of premature infants could be of great interest, as interventions in early life could have a great impact on health in childhood and adulthood. Among nutritional strategies, breast milk has been demonstrated to reduce the burden of respiratory infections in early life [22].

In term infants, human milk is thought to provide a protective effect against wheezing, with a dose-dependent effect conferring greater benefit with a longer duration of human milk feeding [23,24,25]. This effect seems to be more evident in infants of asthmatic mothers and attenuated by milk formula supplementation [26]. Evidence points to a dose-dependent effect of human milk feeding on wheezing, with both duration and exclusivity being associated with a decreased incidence of wheezing in term infants [24,26,27,28]. A meta-analysis by Barchrach and colleagues also demonstrated the protective role that any breastfeeding—regardless of quantity—has on the rates of hospitalization for severe lower respiratory tract disease [29].

The role of human milk on the incidence of respiratory infections in full-term infants has also been investigated and demonstrated in developing countries [30,31].

Nowadays, there is a paucity of knowledge about the role of nutrition in the first months of life of a preterm infant and the subsequent risk of respiratory disorders. For this reason, we aimed to conduct a scoping review to assess the effects of the mode of nutrition on the risk of respiratory infection and wheezing and many aspects of preterm infants’ early-life nutrition. The following research question was formulated: “What is known about the effect of early-life nutrition on the subsequent risk of developing wheezing or respiratory tract infections in preterm infants?”. We aimed to investigate not only the effect of breast milk compared to infant formula, but also the time of the initiation of enteral feeding, the time to reach full enteral feeding, the use of donor human milk or the mother’s milk, and the use of expressed breast milk or breastfeeding.

## 2. Methods

We conducted this scoping review following the PRISMA Extension for Scoping Reviews guidelines (PRISMA-ScR) [32,33,34]. We performed a systematic literature search in April 2022 on the following databases: Web of science, Pubmed, and Embase. The search strings entered are reported in Appendix A. We included any study (RCT, prospective and retrospective cohort study, review, or case report) that analyzed the correlation between “early-life nutrition” and the risk of wheezing and respiratory tract infection in preterm infants (born at less than 37 weeks’ gestation). In particular, we considered the possible effect of human milk (mother’s milk or donor human milk) versus other modes of feeding (standard formula or special formula). Moreover, we considered the method of feeding (gavage, suctioning, breastfeeding, or bottlefeeding) and the time of feeding (the time of the initiation of enteral feeding and the time of reaching full enteral feeding). We considered as “earlylife” anything related to nutrition that happened from birth until the sixth month of life.

Our outcomes of interest were wheezing and respiratory tract infections developed after hospital discharge.

Wheezing was defined based on a physician’s diagnosis; hospitalization for asthma/wheezing; wheezing reported by parents; and the need for bronchodilators. Respiratory tract infections were defined based on a physician’s diagnosis; imaging; hospitalization for RTI; the need for antibiotics; and reports by parents.

We included studies regardless of publication date and language. We excluded narrative and systematic reviews, animal studies, and ongoing studies. We excluded studies focusing on comparisons between different infant formulas, on the mother’s diet or therapy during or after giving birth, on complementary feeding or nutrition after six months of age, and on term infants. Additional studies were identified by screening the reference lists of the screened articles.

Two authors systematically screened all titles and abstracts obtained from the searches of the databases and consequently assessed the full texts of all the potentially eligible studies. We resolved any disagreements by discussion or by consultation with a third author. Afterward, two independent reviewers extracted study characteristics using a predefined charting form that was further refined. The relevant information extracted included: author/year, country of publication, article type, study design, participant characteristics, intervention, and outcomes.

## 3. Results

**Figure 1:**
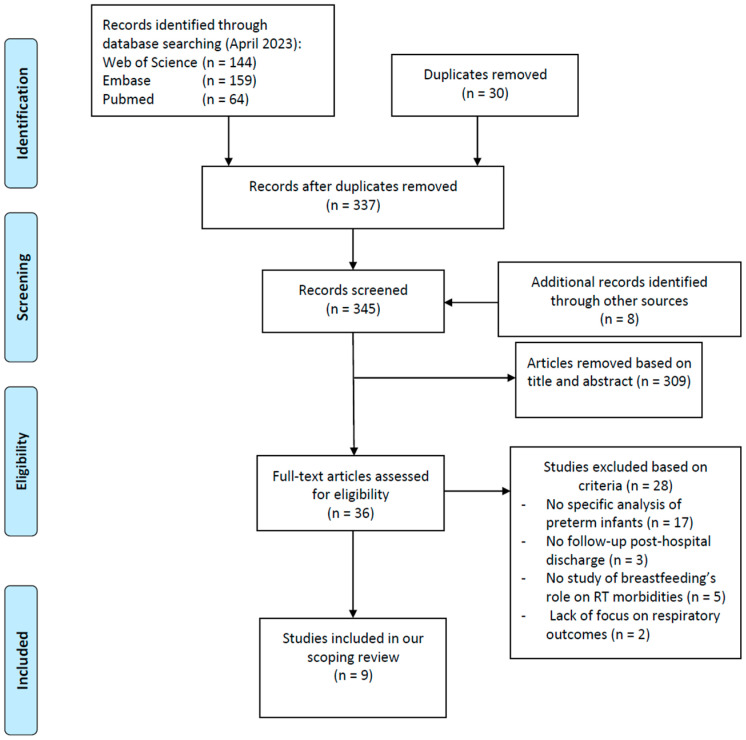
PRISMA 2009 research method flow diagram.

The search generated 337 references, and by screening the reference lists 8 more articles were retrieved, leading to a final count of 345 publications (Figure 1). After title, abstract, and full-text screening, we included nine studies. The characteristics of all included studies are reported in Table 1, and the methodological information (methods, sample size, age at intervention, and outcome assessment) for each article can be found in Table 2.

### 3.1. Role of Early-Life Nutritionin Risk of Hospitalizationfor Respiratory Morbidities

In our literature review, we found one observational study that recognized the role of maternal milk in both wheezing and respiratory infections.

In 2021, Jain and colleagues conducted a prospective observational study at a tertiary-level NICU in India to determine the incidence of respiratory morbidities requiring hospitalization during the first year in preterm neonates and which maternal or neonatal characteristics and environmental factors could influence the risk of readmission [39]. The respiratory morbidities were defined as bronchiolitis (signs and symptoms including a viral upper respiratory prodrome, followed by increased respiratory effort and wheezing); pneumonia (fever, tachypnoea, chest retractions, and with X-ray changes); and wheezing-associated lower respiratory tract infections. They enrolled a total of 344 infants (mean GA 32.7 +/− 2.5 weeks) and followed them up at 1, 3, 6, 9, and 12 months; of these, 58 neonates were hospitalized within the first year of life due to respiratory morbidities. The authors found that a lack of exclusive breastfeeding in the first 6 months of life was associated with a significantly higher risk of hospitalization (adjusted odds ratio of 4.7): 42% of the infants who were readmitted were exclusively breastfed for 6 months, while the portion of breastfed infants went up to 85.6% in the group of children who were not admitted. The association was confirmed by both univariate and multivariate analysis.

Among the other factors investigated, gestational age < 32 weeks, birth weight < 1500 g, APGAR score < 7 at 5 min, a need for surfactant therapy at birth, the presence of hemodynamically significant PDA, a need for respiratory support at birth, and lower socioeconomic status were significantly associated with an increased risk of readmission.

On the other hand, one study found conflicting results upon the analysis of outcomes based on gender [40]. Klein et al. investigated whether the effect of breastfeeding on susceptibility to severe acute lung disease among preterm infants was different for girls and boys. They enrolled a cohort of 119 preterm VLBW infants, with a birth weight below 1500 g and an average gestational age of 29.8 weeks. They divided the population into breastfed (not exclusively) for over or less than 4 months. Acute respiratory infection was defined as the sudden onset of wheezing, rhinorrhea, pharyngitis, cough, retractions, or crackles with or without fever. The main outcome variable investigated was severe acute lung disease, which was defined as a need for re-hospitalization—increased oxygen requirements and the development of respiratory distress. The authors found that breast milk had a statistically significant protective role against re-hospitalization for respiratory morbidities in girls (*p* = 0.003), but this significance did not persist for boys (*p* = 0.598). Therefore, this study was able to identify non-breastfed infant girls as the group most susceptible to severe acute lung diseases. In fact, all but one non-breastfed girl experienced an LRTI.

### 3.2. Role of Early-Life Nutrition in Wheezing

Regarding wheezing itself, a total of three studies did not find a significant correlation between human milk feeding and wheezing. One of them did not find any difference between term and preterm formula and between various durations of the consumption of human milk [42].

In the 1990s, Elder et al. followed a cohort of Australian very premature infants (<33 weeks) in order to identify the prevalence and risk factors for recurrent wheezing treated with bronchodilators in the first year of life [37]. Data on feeding methods were collected with a monthly diary, which also documented the respiratory symptoms. The total duration of breastfeeding after discharge was classified as none, <1 month, 1–2 months, 3–6 months, and >6 months. They included 648 preterm infants and 532 were followed up for the first year of life. Among these, 154 (29.3%) were artificially fed, 49 (9.3%) breastfed for <1 month, 53 (10.1%) breastfed for 1–2 months, 126 (24%) breastfed for 3–6 months, and 143 (27.2%) breastfed for more than 6 months.

The authors confirmed, as already known, that the incidence of recurrent wheezing was higher in infants born preterm than in term ones (14.5% versus 3.0%). Moreover, breastfeeding for 1 or more months after discharge appeared to be protective against recurrent wheezing when compared to no or <1 month breastfeeding in a univariate analysis (OR 0.48, CI 0.30–0.79). This association did not persist in the logistic regression analysis, after controlling for all significant univariate risk factors (adjusted OR 0.74, CI 0.43–1.29).

In their study, Elder and colleagues also evaluated the relation between maternal smoking and the duration of breastfeeding after discharge and wheezing. They found that even though breastfeeding did not have a significant impact on wheezing rates in the overall cohort, when considering children of heavy-smoker mothers specifically, prolonged breastfeeding (>6 months) seemed to have a protective effect.

In the same decade as Elder, a randomized prospective study was published on the effect of the early diets of preterm infants on the development of allergic diseases [42]. The diet was assigned from birth to the time of discharge or until the achievement of 2000 g of weight. Patients were randomized in two parallel clinical trials: in trial A, they were assigned to receive either banked or donor breast milk (*n* = 227) or a preterm formula (*n* = 219) (as their sole diet or as a supplement to their mother’s expressed breast milk when this was insufficient); in trial B, they were assigned to term formula (*n* = 167) or preterm formula (*n* = 164) (alone or in addition to breast milk when this was insufficient). Wheezing or asthma were diagnosed by a collection of information at 18 months follow-up. Observers were blind to the infants’ initial diet. Similarly to Elder, Lucas et al. also did not find any protective effect of exclusive breast milk against the development of wheezing (OR 1.0, CI 0.6–1.5), nor any differences in the incidence of wheezing or asthma between the term formula group and the preterm formula group.

Moreover, the authors conducted a subgroup analysis comparing 47 infants who received human milk alone for 8 weeks or more with those who received it for less than 8 weeks and one who never received it. In this case, they again did not find any differences in the respiratory outcomes.

In 2021, Hsu and colleagues conducted a prospective study in order to determine how early-life factors could affect the development of wheezing in premature neonates [38]. This study included infants born at 34 weeks of gestational age or less who had a history of respiratory support after birth—nasal continuous positive airway pressure, nasal intermittent mandatory ventilation, or mechanical ventilation. The study population comprised 125 infants, who were divided into wheezing and non-wheezing groups. Wheezing was defined as diagnosis by a pediatrician or as the use of asthma medications—inhaled selective beta2 agonists or inhaled corticosteroids—at least twice a year for one year, or the use of leukotrienes modifiers for at least 1 month. Patients were followed-up for 3 years. The wheezing group comprised 19 patients, while the remaining 106 were included in the non-wheezing group. Breast milk feeding was defined solely at the discharge of the neonate. The investigators found a higher rate of exclusive breastfeeding in the wheezing group (*p* = 0.012), although this association was found to be non-significant after multivariable logistic regression.

On the other hand, a more recent study was able to demonstrate a significant protective role of breastfeeding on wheezing in preterm infants. This study was conducted in 2022 by Benson et al., who aimed at determining the association between human milk exposure and recurrent wheezing in preterm Black infants [35]. The study comprised 257 patients, born between 28 and 36 weeks of gestation. They divided the study population into: exclusive human milk (regardless of the route of administration), partial human milk, and exclusively formula fed. Infants were assigned to one of these categories based on their feeding practice at the third month of corrected age. The authors then followed up the patient population for one year, and they found that infants receiving formula milk exclusively were more likely to be hospitalized for respiratory illnesses compared to those receiving any quantity of human milk (OR 0.12 CI 0.02–0.79); no infant receiving exclusive human milk was hospitalized. As for the incidence of wheezing in the first year of life, exclusive human milk feeding at three months was found to be associated with a decrease in the total number of episodes for preterm Black infants.

### 3.3. Role of Early-Life Nutrition in Respiratory Tract Infections

As far as respiratory infection is concerned, three studies were able to positively correlate breastfeeding with a lower incidence of disease.

In 2002, Blaymore and colleagues conducted a study to investigate the role of early-life nutrition on respiratory infections in premature infants [36]. In particular, they wanted to study the anti-infective properties of human milk beyond the special care nursery, throughout the first year of life. A total of 39 patients were enrolled and followed up at 1 month after discharge and at 3, 7, and 12 months of corrected age. Gestational age was similar in the two groups (29 weeks in both the human milk and formula groups). At the time of discharge, 24 newborns were receiving any amount of maternal milk—only 5 human milk exclusively—and 15 received exclusively formula. Among the human milk group, 7 infants no longer received human milk between 1 month post-discharge and 3 months of corrected age; between 3 and 7 months of corrected age, 15 infants (out of 21—71%) stopped receiving any amount of breast milk; and at 1 year, only 8 infants were continuing breastfeeding (3 of whom were exclusively breastfed).

The authors registered the incidence of respiratory infections in terms of days of upper respiratory symptoms (including runny nose, cough, or both). Infants who received breast milk were found to have fewer days of upper respiratory tract symptoms compared to the formula-fed newborns during their first month post-discharge (*p* = 0.016) and at 7 months of corrected age (*p* < 0.025).

The difference in the incidence of upper respiratory tract symptoms was found to be non-significant at 3 months of corrected age (*p* = 0.06) and at 1 year. Furthermore, the authors found no significant differences in the number of days of bronchiolitis in the breastfed versus formula-fed group.

Other factors were investigated—such as birth weight, gestation, gender, maternal age, parental tobacco use, number of siblings, and day-care attendance—but the results found no differences between these groups.

Ofman and his colleagues analyzed the incidence of various risk factors on lower respiratory tract infection (LRTI) and consequent respiratory failure (RF) in premature children in a developing country, namely Argentina [43]. They enrolled 664 premature infants who were re-hospitalized due to severe LRTI—defined as the sudden onset of cough, tachypnoea, wheezing, retractions, crackles, and the need for oxygen supplementation due to acute respiratory symptoms. Among the participants, 60 children experienced RF—defined as an episode requiring mechanical ventilation or resulting in death during hospitalization for LRTI—and 15 died. Many risk factors were investigated, both biological and clinical, but also socioeconomic and environmental. As far as breastfeeding is concerned, the authors found that never being breastfed significantly increased the risk of respiratory failure.

Conversely, one study failed in demonstrating a protective effect of breastfeeding on the risk of hospitalization for bronchiolitis. Recently, Lanari et al. evaluated the risk factors for bronchiolitis hospitalization during the first year of life in children of different gestational ages in Italy [41]. In the population analyzed, both term and preterm infants (>33 weeks) were included, and the comparison was carried out between three cohorts: 33–34 weeks gestational age, 35–37 weeks, and term infants (>37 weeks). In this case, infants receiving RSV (respiratory syncytial virus) prophylaxis with palivizumab were excluded. Follow-up was carried out with two phone interviews with the parents by a trained interviewer. Early-life nutrition was analyzed at both hospital discharge and follow-up, classifying patients as “never breastfed” or “ever breastfed”, with this last category divided into “exclusively breastfed” or receiving a mix of “mother’s and artificial milk”. Breastfed was considered as feeding with maternal milk either from the breast or bottle.

Two thousand two hundred and ten infants were included and followed up until one year of age; among them, 120 (5.4%) were hospitalized for bronchiolitis, 95 of whom were preterm (85%). There was an increasing incidence of hospitalization from lower gestational ages to term (7.3% 33–34 weeks, 5.3% 35–37 weeks, 3.5% term) and, according to univariate analysis, the risk estimate for hospitalization increased 2- and 1.5-fold for those born at 33–34 and 35–37 weeks’ gestational age compared to term infants. While analyzing risk factors for hospitalization, Lanari and co-authors found that a lack of breastfeeding was associated with a significantly higher risk of bronchiolitis admission according to univariate and multivariate analysis in the general cohort (preterm and term). This effect was not confirmed at the subgroup analysis on the group of preterm babies born at 33–34 weeks’ GA (gestational age).

Regarding the use of antibiotics for RTI, Jain et al. [39] defined the use of intravenous antibiotics as a criterion of hospital infant admission for RTI. Hsu et al. [38] recorded the use of maternal prenatal antibiotics among the two cohorts. Conversely, Lanari et al. [41] described the use of antibiotic therapy during neonatal hospitalization and found that babies who underwent this therapy had a higher risk of wheezing in the univariate analysis (HR 2.0 (1.1–3.5)) but not in the multivariate analysis. None of these authors reported the type of antibiotic used.

## 4. Studies Excluded after Full-Text Screening

Three studies were excluded after the full-text screening because they included a mixed population of term and preterm infants, but subgroup analysis was not found [44,45,46].

These three studies were all able to demonstrate that exclusive breastfeeding provided protection from acute respiratory tract infections, therefore also lowering the rates of hospitalization for respiratory morbidities, in the overall population. As previously mentioned, while the three research groups all took into consideration preterm infants as well as full-term ones, the role of breastfeeding in the premature population specifically was not investigated.

Another study was excluded as well, as the authors investigated various infectious outcomes solely during hospitalization and did not follow up patients after discharge [47]. Among the infectious outcomes recorded, pneumonia—defined as respiratory distress not attributable to other causes, combined with radiographic findings suggestive of pneumonia and a positive tracheal culture—was found to be significantly more common in preterm infants receiving formula milk compared to the maternally fed group.

A group of interesting studies was excluded because they did not consider the effect of early nutritional intervention such as oropharyngeal colostrum on the risk of infection after hospital discharge, but only during hospitalization [48,49].

## 5. Discussion

The present review included nine articles that investigated the relationship between early-life nutrition and respiratory outcomes in the preterm population. The aim of our study was to evaluate the effects (mode and timing) of nutrition in the first days and months of life on the risk of respiratory infections and wheezing in preterm infants, based on the current evidence, and to identify knowledge gaps for future research in this field.

In our review, we found a degree of heterogeneity among the definitions of breastfeeding that each study adopted, especially in terms of duration. Two studies defined the feeding groups based on the nutritional state at the time of discharge from the hospital, thereby not investigating the overall duration of breastfeeding [36,42]. Two studies took into consideration different groups based on whether infants ever received breast milk [43] or ever received formula milk [38], regardless of the quantity or duration of breastfeeding. One study adopted 6 months as the cut-off to divide the patient population into exclusively breastfed and not exclusively fed with maternal milk [39]; another study set this cut-off at 3 months [35]; a third one set the cut-off at discharge and at the time of the acute infectious episode [40]. Lastly, one study assessed the nutritional status at three separate moments: upon hospital discharge, at the end of the epidemic RSV season, and at 1 year of life [41]. The most exhaustive classification was provided by Elder et al. [37], who in their study classified the duration of breastfeeding after discharge into none, less than 1 month, 1–2 months, 3–6 months, and over 6 months.

Regardless of the variability described above, a common theme was that a longer breastfeeding duration was directly correlated to decreased respiratory morbidity.

A detailed analysis on the effects of maternal milk on short-term nutritional and infectious outcomes was performed by Cortez et al. in a prospective observational study [47]. Days until full enteral nutrition and the duration of parenteral nutrition were found to be lower in the subgroup of infants who received maternal milk compared to the preterm formula infants, as was the incidence of pneumonia during hospitalization. The authors were also able to provide an objective definition of “exclusive” maternal milk in terms of quantity: preterm infants were said to be fed exclusively maternal milk if they did not receive any amount of formula milk or if they received minimal supplementation, meaning < 5% of the cumulative enteral volume. This study was not included in our review as the authors did not analyze respiratory outcomes after discharge, but the detailed description and classification of early-life nutrition could be considered as a model for future research on this topic.

Although the duration of breastfeeding and its implications for the infant’s health have been the subject of many studies, the role that even a small quantity of human milk in the first days of life could play in respiratory outcomes has been scarcely studied. While this aspect has limited applicability in late preterm infants, it may carry significant relevance for very premature newborns, especially below 32 weeks of gestational age, as they may be unable to receive breast milk per os for many days while clinically unstable [50]. Following this line of thought, in 2009, Rodriguez first proposed the concept of the oropharyngeal administration of colostrum [51]. This practice consists in placing a small quantity of colostrum directly onto the oral mucosa, with the idea that the liquid and its components are absorbed by the mucous membrane. It is a simple and feasible procedure that does not carry any additional risks for the preterm infant [52]. Rodriguez suggested that the oropharyngeal administration of colostrum could be a potential option to expose premature infants to the benefits of colostrum, especially in the first weeks of life when they have the highest risk for infection. After this, Ma et al. conducted a systematic review and meta-analysis on the role of oropharyngeal colostrum in preventing complications of prematurity in VLBW infants, including the risk of the development of ventilator-acquired pneumonia (VAP) [48]. They included VAP, together with NEC, BPD, late-onset sepsis, and death, as the primary outcomes of their study; as for VAP, they found that oropharyngeal colostrum administration was associated with a significant reduction in the incidence of this disease. On the other hand, in the same year, another randomized controlled pilot study including 200 preterm infants did not find any protective effect of early oral colostrum on the development of VAP [49]. Recently, the European Society for Pediatric Gastroenterology, Hepatology, and Nutrition (ESPHGAN) stated that no clear clinical benefits have consistently been proven in high-resource settings, and there are therefore no current data to recommend the routine administration of buccal colostrum to reduce morbidity or mortality, although there may be wider behavioral effects and other benefits [53]. Further research may focus on the short- and long-term effects of the early administration of maternal milk. Since the cited trials were conducted in the recent past, following these study populations could be of great interest to better understand if this early administration of colostrum could have an impact on wheezing and RTI later in life.

Of note, only two out of the nine included studies specified the type of formula used by neonates who were not exclusively breastfed. In particular, in the study of Blaymore et al. [36], preterm infants were given a preterm formula (22 kcal/oz) until they reached 6 months of CA, when they were passed to standard formula (20 kcal/oz), while in the study of Lucas et al. [42] the effect of human milk was compared to preterm formula with a 20 g/L cow protein concentration and a ratio of casein to whey of 40:60.

The heterogeneity previously described for breastfeeding definitions was also found in the outcomes investigated by each study. A total of two studies focused on respiratory infections [41,43], while four studies focused on wheezing [35,42]; two studies included in their outcomes respiratory morbidities, therefore including both entities [39,40]. One study investigated days spent with upper respiratory tract symptoms—runny nose and/or cough [36]. It is worth mentioning that the studies often took into account several interventions and outcomes, but in our review we considered specifically respiratory morbidity.

In regard to wheezing development, there is evidence that a family history of asthma can predict persistent childhood wheezing among male children [54].

Six out of nine studies included in this review took into account the familiar history of respiratory morbidities—specifically, asthma, bronchial hyperreactivity, and wheezing—and atopic diseases. In particular, Benson [35], Hsu [38], and Klein [40] collected data on the parental history for each patient, but they did not perform specific statistical analyses to assess the impact of a positive family history on the outcomes recorded. On the other hand, Elder and colleagues [37] found that a history of parental bronchial hyperreactivity (BHR) was associated with an increased risk of developing wheeze requiring bronchodilators (OR 1.7 (1.3–2.3) with one parent presenting a positive history for BHR; OR 17.8 (10.1–31.2) for patients with a biparental positive history); the statistical significance of this association was confirmed by an adjusted analysis (OR 2.8 (1.61–4.89)). Lanari [41] instead found that a paternal history of COPD significantly increased the risk of hospitalization for bronchiolitis only according to univariate analysis, while a multivariate analysis did not confirm this association. Lastly, Lucas et al. [42] observed a significant increase in the odds ratio (2.3) for developing one or more allergic reactions in infants with a positive family history of atopy receiving preterm formula.

Furthermore, there was also substantial variability in terms of the age at follow-up for outcome registration. Indeed, six out of nine studies [35,36,37,39,40,41] decided to consider respiratory morbidities until 12 months of age or corrected age. Three studies followed up patients until 18 months of corrected age and two and/or three years of age, respectively [38,42,43].

Overall, the nine studies that were included in this review were not able to provide a definite answer to our expected outcome. Half of the studies [35,36,39,43] demonstrated the statistical significance of breastfeeding in protecting preterm infants from respiratory outcomes. On the other hand, four studies did not find that breastfeeding had a significant protective effect against respiratory morbidity—specifically wheezing [37,38,42] and hospitalization for bronchiolitis [41]. One of the studies, as previously described, found conflicting results depending on the gender of the infant [40]. There was no significant difference in the study designs that could account for the different outcomes obtained. The sample sizes varied for each study—from 39 patients in the study by Blaymore et al. up to 2210 infants, among whom 1504 were preterm, enrolled by Lanari and colleagues—but the patient population always comprised premature infants exclusively, with the exception of Lanari [41], who included term infants as well, up to 37 weeks of gestational age. Six out of the nine studies performed an adjusted analysis for confounding factors, to better clarify the specific role of the nutritional mode in the outcomes recorded [35,37,38,39,40,41]. These studies were found to be evenly distributed between the two groups of studies, i.e., those that did and those that did not find a significant correlation between breastfeeding and respiratory illness; therefore, whether external factors could influence the relationship between the two factors investigated could not be determined.

Preterm birth comes with an enormous spectrum of consequences, impacting the neonate from 360 degrees. One of the systems most deeply affected by prematurity is the immune system, with reduced innate and adaptive immunity [55]. Even in full-term neonates, the immune system is immature at birth, which directly relates to an increased risk of infections in newborns, infants, and children [56].

An important component of a newborn’s immune system is constituted by the mucosal immune system, whose development is highly dependent on bioactive factors contributed by breast milk [57]. Among these, secretory immunoglobulins (sIgA) are active against a variety of viruses (respiratory syncytial virus, enteroviruses, herpesviruses, rubella, reovirus, and rotavirus); bacteria (*E. coli*, *Shigella*, *Salmonella*, *Campylobacter*, *Vibrio cholerae*, *H. influenzae*, *S. pneumoniae*, *Clostridium difficile*, *C. botulinum*, and *Klebsiella pneumoniae*); the parasite Giardia; and the fungus Candida albicans. Other bioactive factors include lactoferrin, lysozyme, casein, oligosaccharides, glycoconjugates, and lipids [58]. Lactoferrin is able to block the adsorption and penetration of viruses and the adhesion of bacteria (by chelating iron) and to modulate the activity of monocytes. Lysozyme acts on the bacterial cell wall, causing its lysis; inhibits endotoxins activity; increases IgA production; and is involved in macrophage activation. Casein negatively affects the adhesion of bacteria on the epithelium. Oligosaccharides are the main carbohydrate component in breast milk, and they primarily contribute to energy production. Glycoconjugates bind specific bacterial (*V. cholerae*) and viral ligands (*rotavirus*). Lipids include free fatty acids (FFAs), which have a lytic effect on viruses and an antiprotozoal effect, specifically against Giardia. Lastly, immune modulating agents, especially cytokines, growth factors, IL-10, and IFN-gamma, are contained in human milk and act at the mucosal level [58].

Breast milk has an overall anti-inflammatory effect: even though it contains phlogistic agents, these are present in only limited amounts and are neutralized by other soluble receptors that are also present in human milk. Additional elements that contribute to human milk’s anti-inflammatory action are antioxidants, antiproteases, IL-10, IFN-gamma, cytokine receptors and antagonists, sIgA (as seen previously), prostaglandins, and others [59,60].

All of the factors present in human milk mentioned above are also able to reduce the risk of asthma and wheezing in later childhood by directly promoting the development of the infant’s immune system [30]. In fact, wheezing may often be triggered by early viral infection with RSV or human rhino virus (HRV), which may lead to increased airway sensitization and inflammation, eventually contributing to airway remodeling [5]. Recently, it has been demonstrated that changes in intestinal microbiota and metabolome could be the main factors responsible for the immunomodulatory effect of breastmilk [61]. Therefore, the influence that the gastrointestinal microbiome has on the lungs and how it can affect respiratory outcomes may be of high interest to understand the effect of early-life nutrition on lung development and outcomes.

As hypothesized by one of the included studies [40], together with humoral immunity and anti-infective molecule transfer, other mechanisms of protection against respiratory infections should be considered. The effect of maternal milk on girls could be explained by the activation of a protective pathway in the immune system that could be gender-specific (as with the role of hormones or differences in Th polarization).

## 6. Limitations, Knowledge Gaps, and Directions for Future Research

All the studies mentioned above adopted categorizations of feeding practices that—even though exhaustive—did not line up precisely with that proposed by the WHO [62], therefore making the results regarding feeding practices only partially comparable to those from other studies.

The definition of breastmilk feeding was mostly determined upon the discharge of the neonate from the NICU, and only one study took into account the nutrition modality during hospital admission [42]. This entails that even if a baby was classified as “formula-fed” after discharge, we cannot be sure whether that child ever received human milk (MM or donor milk) during the hospital stay. This factor could carry great relevance, as the hospital stays of preterm infants can be very long (one or two months); therefore, the beneficial effects of human milk may have a substantial impact during this period, when structural and functional organ development is at a crucial stage.

As for studies on the topic at hand conducted on term infants [63], in our case many studies also had several major methodological flaws and, for example, did not adjust for important confounding variables (e.g., family size, the smoking status of the mothers, BPD, and complications of prematurity during hospital stay) [36,42,43]. This could have compromised the conclusions, as the risk of respiratory morbidities after discharge could be majorly influenced by many factors, both clinical and environmental, that could compromise the development of the respiratory and immunological system. This latter aspect was clearly visible in the study by Elder and colleagues, who highlighted how the association between nutrition and wheezing or RTI appeared to be significant only before adjusting for confounding bias [37]. Another obstacle that we came across while comparing the nine studies that we reviewed was the high heterogeneity in the patient population. Indeed, three studies included extremely and very preterm infants [36,37,38], with one study including only moderate to late preterm infants [41], and three studies included all infants born prematurely without GA limits [35,39,43]. Lastly, two studies included preterm neonates based on birth weight, one study included infants with a birth weight below 1850 g [42], and another study set 1500 g as the cut-off for inclusion [40]. In our opinion, this was a crucial point when interpreting the results because, as we already mentioned, infants born at the lowest gestational ages carry the highest risk for respiratory morbidities and long-term respiratory outcomes.

Furthermore, an additional methodological flaw that we encountered was that many of the included studies collected information on breastfeeding post-discharge and on respiratory symptoms via parental reports—either phone calls or home diaries. For this reason, the data may be inaccurate, therefore under- or overestimating the incidence of the recorded outcomes, depending on the subjective parental concern.

Lastly, another aspect that we encountered in our review of the study results was the lack of randomized allocation to early diets. Only one study from 1990 [42] randomly assigned infants to either the exclusive human milk group (their own mother’s milk plus donor milk or exclusively donor) or the preterm formula group (in addition to their own mother’s milk or as their sole diet). The authors found no difference between the two populations, but it is important to note that in both groups there were infants receiving some human milk; therefore, the results could have been distorted by the potential protective effect of even a small amount of human milk ingested by part of the control group. Nowadays, this type of randomization into maternal and formula milk groups would be unethical, given the recognized biological superiority of human milk compared to artificial milk in preterm infant feeding practice [53].

Preterm infants are known to have lower breastfeeding rates than term infants, with those born at the youngest gestation ages being the least breastfed [40,64,65]. However, we know that breastfeeding a preterm infant requires additional knowledge, support, and equipment compared to breastfeeding term newborns. While it seems that policies promoting breastfeeding have not had a detectable effect on lung function—measured in terms of asthma and wheezing—in adolescence [66], the effects of these policies have been investigated only among a healthy term population; patients with a higher risk of respiratory morbidities—such as premature infants–have not been taken into account. Therefore, we do not know the impact that interventions such as that described above could have on this population.

As a gap in the current knowledge, we found that no study included herein mentioned the effect of nutritional management during hospitalization on the later respiratory outcome (suctioning or gavage, early oropharyngeal colostrum, time to enteral nutrition, and time to full enteral feeding).

Moreover, more research is needed in order to understand the molecular mechanism of the protection provided by breastmilk and its components.

Hence, we came to the conclusion that in order to collect the most reliable data, it is mandatory for future research to adjust the results, in terms of both major comorbidities and external factors, but also in terms of gestational age. Indeed, the burden of comorbidities—especially respiratory—is strictly related to the degree of prematurity [2]. Moreover, patient populations should be classified by future researchers so that different studies are comparable; for example, using the WHO prematurity subcategory definitions (extremely preterm <28 wGA, very preterm 28–32 wGA, and late preterm >32 and <37 wGA) [67]. The definition of feeding practices should be standardized, using the WHO definitions [62], and outcomes should be chosen considering the most reliable means of collection. Hospitalization; the use of bronchodilators or antibiotics (by medical prescription); and laboratory, instrumental, or medical diagnosis are the most reliable outcomes that should be evaluated in future research.

## 7. Conclusions

Breastfeeding remains the most recommended mode of feeding in infancy due to the clear health benefits that it provides. However, when taking into consideration the preterm population and respiratory morbidity in terms of respiratory tract infections and wheezing, study results still do not seem to point towards a definite conclusion. According to the current evidence, there seems to be a wide spectrum of risk factors—including feeding methods—that act simultaneously to determine an overall effect on respiratory outcomes and could potentially explain the differences observed in the studies analyzed. Carefully designed studies are needed in order to investigate how different aspects of the early-life nutrition of preterm infants could influence long-term respiratory outcomes.

## Figures and Tables

**Table 1:** Characteristics of included studies.

Study	Country	Population	Principal Aim	Main Findings
Benson 2022 [35]	USA	Preterm infants (28–36 GWk)	Association between human milk exposure at 3 months and recurrent wheezing in the first year of life.	Exclusive MM at 3 months wasassociated with a statistically significant decrease in wheezing episodes. Any MM was associated with a lower risk of hospitalization for respiratory illness.
Blaymore 2002 [36]	USA	Preterm infants (23–34 GWk)	Effect of human milk on upper respiratory tract symptoms.	Breast milk feeding was associated with fewer days of upper respiratory tract symptoms compared to formula feeding.
Elder 1996 [37]	New Zealand, Australia	Preterm infants (<33 GWk)	Prevalence and risk factors for recurrent wheezing treated with bronchodilators in the first year of life.	Breastfeeding appeared to be protective against wheezing in unadjusted analysis. Association not confirmed in the adjusted analysis.
Hsu 2021 [38]	Taiwan	Preterm infants (25–31 GWk)	Association between early-life factors and preschool wheezing in premature neonates.	No protective effect of exclusive breast milk against the development of wheezing.
Jain 2021 [39]	India	Preterm infants (<37 GWk)	Risk factors for respiratory morbidities requiring hospitalization during the first year of life.	Protective role of exclusive breastfeeding for the first 6 months of life on the risk of readmission for respiratory morbidities.
Klein 2008 [40]	Argentina	Preterm infants born < 1500 g	Effect of breastfeeding on severe acute lung disease in girls and boys in the first year of life.	Protective effect of breastfeeding on the risk of hospitalization for severe acute lung disease in girls, no protective effect in boys.
Lanari 2015 [41]	Italy	Mix of term and preterm infants (33–37 GWk)	Risk factors for bronchiolitis hospitalization in the first year of life.	No protective effect of exclusive breast milk against hospitalization for bronchiolitis (on subgroup 33–34 GWk).
Lucas 1990 [42]	UK	Infants born < 1850 g	Effects of early diet on allergic/atopic diseases (eczema, wheezing, food or drug sensitivities) in the first 18 months of life.	No protective effect of exclusive breast milk against the development of wheezing.
Ofman 2020 [43]	Argentina	Preterm infants (<37 GWk)	Risk factors for respiratory failure and fatal lower respiratory tract infection.	Protective role of ever receiving maternal milk on respiratory failure and LRTI.

**Table 2:** Methodological information arranged per author.

Study	Type of Study	Sample Size	Intervention *	Time of Intervention Assessment	Outcomes *	Follow-Up for Outcomes
Benson 2022 [35]	Secondary analysis of existing data	*n* = 275	Exclusive MM; partial (MM + formula); exclusive formula feeding	At 3 months of CA	Recurrent wheezing: report (parental or at study visit) of more than one wheezing episode, occurring at least 2 weeks apart. Secondary outcomes were the total number of wheezing episodes by 12 months of corrected age; respiratory medication use (bronchodilators, ICS, mast cell inhibitors, leukotriene inhibitors, and oral steroids); medically attended respiratory illnesses (resulting in office visit, urgent care or ED visit, or hospital admission).	From discharge to 12 months of CA
Blaymore 2002 [36]	Prospective observational study	*n* = 39	Exclusive formula; MM (with or without formula)	At time of discharge	Upper respiratory symptoms: measured in terms of days of runny nose, cough, or both.	1 month after discharge and at 3, 7, and 12 months CA
Elder 1996 [37]	Prospective observational study	*n* = 648	MM > 1 month; MM < 1 month (regardless of exclusiveness)	At time of discharge, then monthly for six months after discharge.	Recurrent wheezing treated with bronchodilators: reporting two or more episodes of respiratoryillnesses associated with wheezing for which the general practitioner or pediatrician prescribed a bronchodilator.	12 months CA
Hsu 2021 [38]	Prospective observational study	*n* = 125	Exclusive breast milk feeding; any formula milk	Not specified	Wheezing: diagnosis by a pediatrician or the use of asthma medications—inhaled selective beta2 agonists or inhaled corticosteroids—at least twice a year for one year, or the use of leukotriene modifiers for at least 1 month.	From discharge to 3 years
Jain 2021 [39]	Prospective observational study	*n* = 344	Exclusive BF for 6 months	6 months	Hospitalization due to the following respiratory morbidities: a. Bronchiolitis—a constellation of clinical symptoms and signs including a viral upper respiratory prodrome, followed by increased respiratory effort and wheezing in young infants. b. Pneumonia—the presence of fever, tachypnea, chest retractions, inability to drink, or central cyanosis along with chest X-ray changes. c. Wheezing-associated lower respiratory tract infection was defined as tachypnea, dyspnea, and wheezing on auscultation with or without fever.	Follow-up visits at 1, 3, 6, 9, and 12 months.
Klein 2008 [40]	Prospective observational study	*n* = 119	Breastfeeding (yes/no)	At hospital discharge, at monthly visit	Severe acute lung disease: need for rehospitalization (determined on the basis of changes in baseline oxygen requirement and the development of respiratory distress).	Follow-up visits monthly until 12 months of corrected age.
Lanari 2015 [41]	Prospective observational study	*n* = 2210 infants *n* = 1504 preterm (*n* = 737 33–34 GWks; *n* = 767 35–37 GWks)	Never breastfed; exclusively breastfed; MM and artificial milk	At hospital discharge, at the end of the epidemic RSV season (end of March), at 1 year of life	Hospitalization/death for bronchiolitis: defined according to the hospital discharge form with the ICD-9 codes 466.1 (acutebronchiolitis).	From discharge until 12 months of age.
Lucas 1990 [42]	Randomized prospective trial	*n* = 446	HM vs. preterm formula; term formula vs. preterm formula; HM > 8 weeks vs. HM < 8 weeks vs. only formula	From birth until discharge or achievement of 2000 g	Recurrent wheezing: intermittent increase in airway resistance, presenting as a cough or wheeze that could be reversed, largely or completely, by bronchodilators or steroids. Other respiratory conditions characterized by noisy breathing (for example, mucous rattling) were excluded.	From discharge until 18 months of CA.
Ofman 2020 [43]	Prospective, population-based, cross- sectional, multicenter study	*n* = 664	Never BF (no information on exclusiveness)	Not specified	Severe LRTI:sudden onset of cough, tachypnea, wheezing, retractions, and/or crackles with or without fever, and either an oxygen saturation <93% at rest when breathing room air, or arriving to the emergency room (ER) receiving oxygen supplementation due to acute respiratory symptoms. Respiratory failure: defined as an episode (1) requiring mechanical ventilation (excluding noninvasive ventilation or continue positive airway pressure devices) or (2) resulting in death during hospitalization due to LRTI.	Under 2 years.

ICS: inhaled corticosteroids, ED: emergency department. * The studies often analyzed several interventions and outcomes, but here we just reported those included in our review.

## Data Availability

Data sharing not applicable.

## Abbreviations

BPD	Bronchopulmonary dysplasia
CA	Corrected age
FEV1	Forced expiratory volume 1 s
GA	Gestational age
HM	Human milk
LRTI	Lower respiratory tract infection
MM	Maternal milk
NEC	Necrotizing enterocolitis
RTI	Respiratory tract infection
RCT	Randomized controlled trial
RSV	Rhino syncytial virus
VAP	Ventilatory acquired pneumonia
VLBW	Very low birth weight

## Appendix A

Web of science: ((premature infant) OR (premature newborn) OR (preterm infant) OR (preterm newborn) OR (prematurity)) AND ((respiratory tract infection) OR wheezing OR (bronchial spasm)) AND ((feeding OR nutrition OR breastfeeding OR (bottle feeding) OR breastmilk)).

Embase: (‘respiratory tract infection’/exp OR ‘respiratory tract infection’) AND ‘prematurity’/exp AND ‘infant feeding’/exp.

Pubmed: (((“Respiratory Tract Infections”[Mesh]) OR ((“Respiratory Sounds”[Mesh]) OR “Bronchial Spasm”[Mesh])) AND (((“Diet, Food, and Nutrition”[Mesh]) OR “Feeding Methods”[Mesh]) OR “Feeding Behavior”[Mesh])) AND (“Infant, Extremely Premature”[Mesh] OR “Infant, Premature”[Mesh]).
